# Amnion Epithelial Cells as a Candidate Therapy for Acute and Chronic Lung Injury

**DOI:** 10.1155/2012/709763

**Published:** 2012-04-11

**Authors:** Ryan J. Hodges, Rebecca Lim, Graham Jenkin, Euan M. Wallace

**Affiliations:** The Ritchie Centre, Monash Institute of Medical Research and Department of Obstetrics and Gynaecology, Southern Clinical School and Monash Medical Centre, Monash University, 246 Clayton Road, Clayton, VIC 3168, Australia

## Abstract

Acute and chronic lung injury represents a major and growing global burden of disease. For many of these lung diseases, the damage is irreparable, exhausting the host's ability to regenerate new lung, and current therapies are simply supportive rather than restorative. Cell-based therapies offer the promise of tissue regeneration for many organs. In this paper, we examine the potential application of amnion epithelial cells, derived from the term placenta, to lung regeneration. We discuss their unique properties of plasticity and immunomodulation, reviewing the experimental evidence that amnion epithelial cells can prevent and repair lung injury, offering the potential to be applied to both neonatal, childhood, and adult lung disease. It is amazing to suggest that the placenta may offer renewed life after birth as well as securing new life before.

## 1. Introduction

Chronic lung diseases, in both children and adults, are leading causes of morbidity and mortality worldwide, estimated to account for about 10% of global mortality [1]. It has been estimated by the World Health Organization that, by 2030, chronic lung disease, mainly caused by tobacco smoking, occupational irritant exposure and pollution, will become the third most common cause of death worldwide [1]. However, mortality is just the tip of the iceberg. A recent economic analysis of the burden of chronic lung disease in Australia revealed that almost 1 in 5 adults aged 40 or older have chronic obstructive pulmonary disease (COPD) to some degree, with half of these individuals having advanced disease [2]. The estimated financial cost of COPD in Australia in 2008 was nearly $9 billion. Further, chronic lung disease does not only affect adults. About 1 in 80 children aged under 10 suffer morbidity from COPD. In particular, over recent decades advances in perinatal care have greatly improved the survival chances of very preterm babies, principally through the reduction of acute respiratory distress syndrome (RDS) by antenatal corticosteroids and postnatal surfactant therapies [3, 4]. However, almost a third of these survivors develop chronic neonatal lung disease, so called bronchopulmonary dysplasia (BPD), a disease with the consequent long-term burdens of childhood respiratory dysfunction and neurodevelopmental delay [5]. Unfortunately, both neonatal BPD and adult COPD have an important feature in common. Neither have an effective treatment.

Accordingly, together, these childhood and adult chronic lung conditions represent a significant and growing burden of disease for which there is no targeted intervention that might restore lung function and thereby reduce morbidity and mortality. However, while the causes of childhood and adult COPD differ, the fundamental lung injury is similar—chronic inflammation, fibrosis, and scarring [6–8]—and the clinical end result—loss of functional lung tissue—is identical. As such these chronic lung diseases may be amenable to regeneration, which may be afforded by cell-based therapies. In this paper we review the recent advances in the application of placenta-derived cells as a potential therapy for human lung disease. Specifically, we will review the unique properties of amnion, the effect of amnion cells on different models of lung injury and explore the likely mechanisms of action of amnion cells in lung repair with a view to human clinical trials.

## 2. Unique Therapeutic Properties of Amnion Epithelial Cells

At the first international workshop on placenta-derived stem cells, convened in Brescia, Italy in 2007, two key properties of placental cells that make them attractive for regenerative medicine were highlighted: *plasticity* and *immunomodulation *[9]. Human amnion epithelial cells (hAECs) are a subset of placental-derived stem cells that display both of these key features and, perhaps, possess advantages over the other populations of stem cell-like cells in the placental tissues. First, the amnion itself is derived from the embryonic epiblast prior to gastrulation. This is important because cells derived from the epiblast prior to gastrulation are thought to retain multipotent *memory* or *plasticity*, reflecting the capability of the epiblast itself to differentiate into the ectoderm, endoderm, and mesoderm of the definitive embryo. Thus, at least theoretically, amnion cells should be capable of differentiation down each primary lineage. Over the past 6 years or so we, and others, have carefully characterized human amnion epithelial cells (hAECs), defining cell types and asking whether they share any transcriptional factors with embryonic stem cells that might confer pluripotentiality. First, cells isolated from amniotic membranes by simple digest are essentially exclusively epithelial cells [10]. These human amnion epithelial cells (hAECs) do not express mesenchymal or haematopoietic cell markers and differ from cells derived from amniotic fluid in early to midpregnancy [10–12]. This distinction between amniotic membrane-derived amnion epithelial cells, and amniotic fluid stem cells is that the former are a pure population of epithelial cells while the latter are a mixed cell population of mesenchymal, stromal, and epithelial cells. This difference is important to keep in mind when assessing possible therapeutic and regenerative medicine applications for each of these cell populations, as will be discussed later. However, while hAECs are all epithelial cells they are still a heterogeneous population of epithelial cells with diverse cell marker expression. Importantly, these cell lineage markers include early “stem cell” markers such as the POU domain, class 5, transcription factor, Nanog homeobox; SRY-2 box, the stage-specific embryonic antigen-4 (SSEA4) [10, 13]. For example, in one report 44% of hAECs expressed SSEA4, 5–15% of cells expressed Oct-3/4, and 5–15% expressed Nanong and/or Sox-2 [10]. Consistent with the expression of such early lineage markers, the differentiation repertoire of hAECs has been confirmed *in vitro* using various techniques (phenotypic, mRNA expression, immunocytochemical, and/or ultrastructural characteristics), demonstrating that hAECs derived from term placental membranes can be successfully differentiated into cardiomyocytic, myocytic, osteocytic, adipocytic (mesodermal), pancreatic, hepatic, lung (endodermal), neural, and astrocytic (neuroectodermal) cells [13, 14]. With regard to the lung, hAECs express thyroid transcription factor or Nkx 2.1 mRNA, one of the earliest lineage markers of the developing lung that is essential for branching lung morphogenesis and type II alveolar cell formation [14].

However, while hAECs express many markers of early stem cells, they are not omnipotent like embryonic stem cells. Indeed, there are a number of key differences between hAECs and embryonic stem cells that suggest that hAECS may be more suitable for clinical application. For example, unlike embryonic stem cells (ESCs) and human induced pluripotent stem (IPS) cell lines [15, 16], hAECs do not form teratomas when injected into the testes of mice with severe combined immunodeficiency (SCID) [13, 17] and they maintain a normal karyotype and cell cycle distribution with telomere stability over prolonged *in vitro *passaging [10]. These observations suggest that the more limited pluripotency displayed by hAECs, compared with ESCs or IPS cells, will pose less risks for *in vivo* tumour formation after-transplantation than those other stem cells. Furthermore, hAECs express no, or very little, class IA and class II human leukocyte antigens (HLAs) [13, 18]. In fact, likely reflecting their functions during pregnancy, hAECs express the immunosuppressive human leukocyte antigen G (HLA-G) that confers a degree of immune privilege by suppressing NK cells, inducing apoptosis of activated CD8^+^ T-cells and inhibiting CD4^+^ T cell proliferation [18, 19]. Such findings are consistent with promoting maternal tolerance of a fetal allograft (including its membranes) for the nine months of human pregnancy. Such a property might also suggest a low risk of tissue rejection when given therapeutically. So far this indeed appears true. Following xenotransplantation hAECs can survive for prolonged periods in immune competent monkeys, rabbits, guinea pigs, rats, and swine without immunorejection, albeit without confirmed *in vivo* differentiation [19–22]. In one study hAECs transplanted into neonatal swine and rats resulted in human microchimerism in various organs and tissues without immune clearance [19]. Furthermore, hAECs injected into healthy human volunteers did not elicit any clinical signs of acute rejection and recipients did not produce antibodies against HLA antigens [23]. However, while undifferentiated hAECs do not express HLAs, apart from HLA-G, it would appear that as they are made to differentiate, at least *in vitro*, this immune privileged state may be lost. For example, recently we showed that, following differentiation into hepatic and pancreatic lineages, significant numbers of hAECs began to express Class IA, but not Class II HLA [13]. The clinical significance of this finding for future cell transplantation remains unclear but suggests that cells differentiated *in vitro* prior to transplantation may be less suitable for allogeneic use than primary undifferentiated cells. We will revisit this theme later.

## 3. Endogenous Lung Stem Cells

Before any discussion of stem-cell-mediated lung repair, it is useful to distinguish the roles and activities of exogenous stem cells, such as hAECs, from those of resident endogenous lung stem cells. While the very slow natural turnover of lung and bronchial epithelia and the multiple distinct anatomical zones of the lung have made the identification of lung stem cells difficult [24–26], a number of different resident lung stem or progenitor cells have been identified [27, 28]. It is thought that each of these progenitor cell niches provides specific repair mechanisms for the different parts of the respiratory tract and that different injuries may trigger differential responses from the various progenitor cell populations [25, 27, 28]. Specifically, distinct progenitor cells have been identified in the proximal trachea, the bronchi, the bronchioli, the bronchiolar-alveolar junction, and the alveoli [24, 25, 28]. Of these various lung progenitor/stem cells, specialized nonciliated airway epithelial cells called Clara cells, or variant Clara cells, respond to airway injury by replenishing the ciliated epithelium, particularly in the bronchioli and at bronchiolar-alveolar junction, while alveolar type II (ATII) cells are thought to be the principle repair mechanism in the alveoli [25, 26, 28]. With regard to exogenous stem cells and lung repair, the endogenous “resident” lung progenitor/stem cells are likely to be important. As will be discussed later, while it was initially thought that exogenous stem cells affected lung repair by integrating into the damaged epithelium and differentiating into lung cells, it is more likely that the principal mechanism whereby they effect repair is via immunomodulation [29] and by supporting endogenous lung stem cell activity. Indeed, while the lung contains its own population of resident endogenous stem cells, it is thought that their regenerative efforts become exhausted during severe injury, leading to both acute and chronic respiratory embarrassment. It is this feature that makes the lung a particularly receptive organ for exogenous cell therapy.

## 4. Amnion and Models of Lung Injury

The first report of using amnion cells for repairing lung injury was by Carraro and his colleagues [30] who used amniotic fluid stem cells (hAFSCs) obtained from amniocentesis in midpregnancy. They assessed this mixed population of cells that included hAECs, other epithelial cells, and mesenchymal cells with regard to their reparative abilities in two different murine models of lung injury. First, they demonstrated that hAFSCs could engraft into mouse embryonic lung explants *in vitro* and differentiate into a lung-type cell, as evidenced by the expression of thyroid transcription factor 1 (TTF1). Then, to assess the ability of hAFSCs to repair alveolar lung injury they administered the cells to mice following short-term hyperoxia. The hAFSCs migrated to the distal lung and expressed both TTF1 and the type II alveolar cell product surfactant protein C. Next, hAFSCs were administered to mice which had undergone naphthalene lung injury. Naphthalene targets Clara cells in the airways. As with the hyperoxia alveolar injury model, the hAFSCs trafficked to the sites of injury—this time in a bronchoalveolar junction and bronchial distribution rather than the alveoli—and expressed the Clara cell 10 kDa protein. This first report highlighted two key properties of cells derived from amniotic fluid: their ability to track to specific sites of injury and their plasticity to respond *specifically* to the nature of the lung insult itself, differentiating into the cell type that had been injured. Importantly, while the origin of these cells was from a mixed population isolated from amniotic fluid, the cells had been sorted by c-kit positivity—a stem cell marker—and derived from clonal cultures to further select for stem cell-like behaviour. Therefore, it was not surprising that the cells displayed pluripotency. However, in neither of the two injury models was there evidence of amelioration of injury. This suggests that integration and differentiation *in vivo* are not sufficient for exogenous stem cells to effect repair. Furthermore, translating this cell therapy into clinical practice may have some limitations. The cells were derived from amniotic fluid by amniocentesis, selected by c-kit expression and then expanded and purified through clonal isolation. Such a source is not likely to be a ready source of sufficient cells for widespread application because amniocentesis is an invasive procedure that carries a risk of miscarriage. It is unlikely that women will be prepared to expose their pregnancy to such risks for the benefits of others. It is also unlikely that sufficient numbers of cells will be able to be derived for widespread application, although expansion would be feasible.

Utilising placentae from term births is one strategy to circumvent these problems entirely. Cargnoni and her coworkers [31] transplanted a mixed population of fetal membrane-derived cells from the amnion and chorion from both allogeneic and xenogeneic (50% human mesenchymal cells, 50% hAECs) sources, to a murine bleomycin model of adult idiopathic pulmonary fibrosis (IPF). Importantly, unlike the amniotic fluid derived cells used by Carraro and colleagues, the cells used by Cargnoni were primary cells that had not been selected for either c-kit expression or clonal activity. Bleomycin produces many of the histological hallmarks of IPF such as intra-alveolar buds, mural incorporation of collagen, and obliteration of the alveolar space [32]. Both allogeneic and xenogeneic populations of mixed primary cells, administered either systemically or intraperitoneally, mitigated lung fibrosis to a similar extent and markedly reduced neutrophil infiltration, a key prognostic determinant of IPF. Persistence of both cell populations was detected in the lung 14 days after administration confirming engraftment, albeit without confirmed differentiation into a lung phenotype. Indeed, it is unclear if engraftment is even essential for their therapeutic effect. The same group had earlier demonstrated microchimerism in the lung at 90 days only by PCR after intraperitoneal injection of a similar xenogeneic population of cells. That finding suggests that the significance of the engraftment seen in the second study was likely to be modest [19, 31].

Extending these early reports, Moodley and colleagues [14] also used the bleomycin model of lung injury in SCID mice with the aim of examining differentiation of the cells into lung phenotypes in more detail. In this study a pure population of primary, unselected hAECs derived from term placental membranes were used. First, primary undifferentiated hAECs were cultured in small airway growth media (SAGM), known to induce differentiation of umbilical mesenchymal cells and embryonic stem cells into type II pneumocytes [33, 34], to explore whether hAECs could be directed down an alveolar epithelial phenotype lineage. After prolonged culture in SAGM, hAECs appeared to partially differentiate into lung cells, producing surfactant proteins A, B, C, D and displaying ultrastructural evidence of lamellar bodies, an organelle of type II pneumocytes. Further, these cells responded to a glucocorticoid trigger and were capable of secreting surfactant D [14]. None of these features were present in freshly isolated hAECs. Primary, undifferentiated hAECs (lacking surfactant proteins) were also injected intravenously into bleomycin treated SCID mice and were shown to engraft and produce all surfactant proteins [14]. This suggested an ability of primary hAECs to differentiate into lung cells *in vivo*—similar to the observations made by Carraro of selected and purified amniotic derived cells [30]. However, unlike the amniotic fluid derived cells, hAEC administration significantly reduced bleomycin-induced lung fibrosis and inflammation [14]. Specifically, levels of pro-inflammatory cytokines were reduced (monocyte chemo-attractant protein-1, tumor necrosis factor-alpha, IL-1 and IL-6) and anti-inflammatory cytokines were increased (IL-10 and macrophage migration inhibitory factor). Expression of the profibrotic cytokine transforming growth factor-beta was also reduced by hAECs administration. In keeping with these cytokine changes, lung collagen content was reduced, reported to be a consequence of increased action of matrix metalloproteinase-2 and down-regulation of the tissue inhibitors of matrix metalloproteinase-1 and 2 consistent with lung repair. hAECs also seemed to reduce established fibrosis in one small group of mice, with a reduced collagen content confirmed with the delayed administration of hAECs two weeks after the bleomycin insult. This comprehensive study demonstrated that a pure population of primary hAECs derived from term placenta after completion of a pregnancy had the ability to prevent and repair acute lung injury induced by bleomycin. The authors suggested that the cells exerted these affects via modulation of the host response to injury and by *in vivo* differentiation [14].

However, while subsequent studies have confirmed the injury prevention abilities of hAECs, they have cast some doubt on the mechanisms by which hAECs effect this. Using the bleomycin model of lung injury, Murphy et al. [29] advanced the field by the administration of a pure population of undifferentiated hAECs to immune competent, as opposed to immune compromised, mice. In this study, intraperitoneal administration of hAECs 24 hrs after bleomycin administration decreased lung fibrosis, evidenced by reduced collagen deposition and alpha-smooth muscle actin, and decreased lung inflammation and the expression of proinflammatory cytokines [29]. Moving one step further, this was to the first study to demonstrate that the mitigation of structural lung injury by hAECs was associated with a partial restoration of physiological lung function, as assessed by whole body plethysmography. However, in contrast to the previous studies—all performed in immune compromised mice—Murphy and his colleagues were unable to demonstrate any engraftment of hAECs in the lung [29]. Based on this observation, some doubt was cast on the likely mechanism(s) by which hAECs may work.

## 5. Mechanisms of Action of hAECs: Engraftment versus Immunomodulation

It is fair to say that, at present, there remains some uncertainty about the primary mechanism(s) by which hAECs affect lung injury prevention/repair. Specifically, whether *in vivo* engraftment and differentiation are necessary or whether modulation of the host response to injury that then reduces inflammation and fibrosis, either directly or indirectly, is key. At this stage it would appear that the latter is the more likely. Our recent report [29] clearly demonstrated that hAECs can exert a reparative effect without the need for engraftment or differentiation and the work of Carraro and his colleagues showed that engraftment and differentiation *per se* was not sufficient for injury prevention/repair [30]. The apparent inconsistencies between studies regarding whether *in vivo *integration and differentiation of hAECS following injury actually occurs may be explained by the methods used to identify hAECs *in vivo*. Murphy et al. [29] chose *fluorescence-activated cell sorting *(FACS) with gating applied to exclude dead cells to detect only live resident human cells. Previous studies relied on PCR or *in situ* hybridization for human DNA or immunohistochemistry. These latter methods are all unable to discern living from dead cells [14, 30, 31] and so it is possible that those studies were simply reporting dead cells. Of course, this does not explain the *in vivo* identification of surfactant protein-expressing hAECs [14]. Nonetheless, we have suggested that the primary mechanism of injury repair in their study was likely to be paracrine signaling to the surrounding tissues to reduce proinflammatory and profibrotic mediators [29]. This is consistent with previous reports of a beneficial effect of amnion where cellular differentiation has not been confirmed, such as in brain ischaemia, Parkinson's disease, spinal cord injury, myocardial ischaemia, critical limb ischemia, burns and skin wounds [20, 35–39].

The exact identity of antiinflammatory and anti-fibrotic factors that might be released by amnion cells remains to be elucidated. However, the ophthalmology literature has described for some time the beneficial effect of amnion for corneal ulcers mediated through a reduction in HLA Class II antigen presenting cells at the site of injury, reduction in apoptosis and inflammation [40]. This literature suggests hAECs are able to inhibit the chemotactic migration of neutrophils and macrophages to the site of injury, possibly via MIF and suppression of IL-1*α*, IL-1*β*, and proteinase [41], similar to that shown by Moodley et al. [14]. Indeed, hAEC-induced suppression of macrophage and neutrophil migration into the injured lung has been a consistent finding [29, 31]. This is supported by the observation that hAECs decrease macrophage migration *in vitro *[42]. That hAECs modulate macrophage and/or neutrophil migration is likely to be important in the context of lung injury because both macrophages [43] and neutrophils [44] play important roles in mediating such injury. In this regard, very recently we showed that hAECs were unable to mitigate bleomycin-induced lung injury in SP-C knock-out mice [42]. This strain of mouse is known to have deficient macrophage function with macrophages unable to switch from an M1 (pro-fibrotic) phenotype to an M2 (reparative) phenotype. We suggested that this observation was consistent with hAECs exerting their reparative effects via macrophages rather than directly [42].

Of course, hAECs may operate through other mechanisms too. For example, they express the anti-inflammatory IL-1 receptor antagonist, IL-10, collagen XVIII, thrombospondin-1 and all four tissue inhibitors of metalloproteinase (TIMPs) [45]. Apoptosis of leucocytes has been reported and hAECs express apoptosis-inducing genes Fas L, TNF, and TRAIL [41, 46]. Furthermore, there is evidence to support an anti-angiogenenic effect through release of endostatin, TSP-1 and TIMPs and the antibacterial protein lactoferrin [47]. The relative contributions of these pathways to amnion cell-mediated tissue repair will clearly require a considerable amount of working through.

In relation to fibrosis, human corneal and limbal fibroblasts grown on the matrix surface of amniotic membranes displayed marked down regulation of TGF*β*-signalling system with decreased expression of TGF*β*-1, *β*-2 and *β*-3 isoforms and reduced expression of TGF-Receptor II preventing fibroblast activation into myofibroblasts [48]. Furthermore, amniotic membrane is also capable of reversing already differentiated myofibroblasts back into a fibroblast phenotype, which may be particularly useful for ameliorating more established disease [49]. Since TGF-*β* signalling plays a central role in pulmonary fibrosis [50] the ability of hAECs to decrease TGF-*β* signalling and prevent fibroblast activation is likely to be an important effector mechanism in their reparative properties.

## 6. Towards Clinical Trials

Before hAECs can be effectively translated into a future cellular therapy for lung injury a number of questions need resolving. It would be useful if the identity of the anti-inflammatory and antifibrotic signaling pathways at play are characterized. It may be possible that these pathways can be manipulated pharmacologically rather than necessitate cell delivery. In this way, the reparative abilities of hAECs would have been used to develop new drug-based therapies by revealing the key pathways that need targeting. However, such an endeavour will be considerable and more immediate therapy may be afforded by simply administering cells. If this is the case then it will also be necessary to determine the optimum cell type for transplantation, whether that be undifferentiated hAECs, or hAEC-derived partially differentiated lung progenitor cells, or even a mixed cell population. As detailed earlier, the hAECs used in the studies to date have been unselected, primary cells—a heterogeneous population of epithelial cells. It is possible that only a subset of these cells are reparative and that by identifying and purifying those cells more effective regenerative therapies can be developed. Of course, it is also possible that it is the heterogeneous nature of the population that is a key attribute of the therapy, providing diverse cells to undertake diverse roles at different stages of tissue injury-repair-resolution. If this is so then purified subpopulations of cells may prove to be less, rather than more, effective. It is also unknown whether both undifferentiated and partially differentiated cells equally effect immunomodulation. This would be important to define before embarking upon clinical trials. From a pragmatic perspective, regulatory authorities are more likely to approve primary, unmanipulated cells than cells that have undergone extensive purification, selection, and differentiation. However, if differentiated cells are more effective then these should form the basis of future therapies.

With regard to regulatory approval and cell handling, it will also be necessary for future studies to adhere to good manufacturing practice (GMP) processes suitable for clinical use, as has already been described [10, 51], to readily ensure standardization and clinical applicability moving forward. Should hAECs prove useful clinically, such standardization will be a foundation of cell banks. We believe that while autologous use of hAECs may offer a safe first step application, for example in preterm neonates with bronchopulomonary dysplasia [26], ultimately widespread use of hAECs will require the development of biobanks of high quality cells for allogeneic application, most likely in an alpha clinic setting [52]. The clinics and cell banks will be necessary simply because the majority of patients do not have their own amnion cells in storage.

Finally, and possibly most importantly, all of the studies performed to date have administered hAECs very early during the injurious process in the lung. There has been no comprehensive assessment of whether hAECs are able to repair established and long-standing lung injury. This is clearly critical because the most common clinical application of cell therapy for lung disease will be to those individuals with chronic, established and extensive lung injury. This is where the current clinical burden lies and where current therapies desperately fail.

## 7. Conclusion

There is no question that acute and chronic lung disease require novel therapies. Preclinical studies have shown that amnion cells are able to reduce fibrosis and inflammation, and thereby improve lung function. However, several questions remain unanswered, including whether how these cells work, whether there are subpopulations of cells that are most effective, and whether amnion cells are able to repair established disease. We are indeed hopeful that the immunomodulatory concert that is present at the maternal-fetal interface during pregnancy may soon extend long after birth to offer new therapies for sufferers of chronic lung disease.
